# Sex difference in fatigability of knee extensor muscles during sustained low-level contractions

**DOI:** 10.1038/s41598-019-53375-z

**Published:** 2019-11-13

**Authors:** Ryota Akagi, Shinya Sato, Kana Yoshihara, Hideki Ishimatsu, Ryoichi Ema

**Affiliations:** 1 College of Systems Engineering and Science, Shibaura Institute of Technology, 307 Fukasaku, Minuma-ku, Saitama-shi, Saitama, 337-8570 Japan; 2 Graduate School of Engineering and Science, Shibaura Institute of Technology, 307 Fukasaku, Minuma-ku, Saitama-shi, Saitama, 337-8570 Japan; 3 QOL Improvement and Life Science Consortium, Shibaura Institute of Technology, 307 Fukasaku, Minuma-ku, Saitama-shi, Saitama, 337-8570 Japan; 4 School of Management, Shizuoka Sangyo University, 1572-1 Owara, Iwata-shi, Shizuoka, 438-0043 Japan

**Keywords:** Neurophysiology, Skeletal muscle

## Abstract

This study investigated whether the sex difference in fatigability of the knee extensors (KE) is explained by the sex difference in fatigue-induced changes in the shear modulus of one or more muscles of KE in 18 young men and 23 young women. The shear moduli of the resting rectus femoris and medial and lateral vastus muscles (VL) were measured before and after a sustained contraction at 20% peak torque during a maximal voluntary isometric contraction of KE until the endurance limit, in addition to evoked torque and voluntary activation (VA%). The fatigue-induced decrease in maximal muscle strength was more prominent in men than in women. Only the VL shear modulus for men increased after the fatiguing task, and a sex difference was observed in the percentage change in the VL shear modulus before and after the fatiguing task. The fatigue-induced decreased ratio was greater for men than for women in evoked torque, but not in VA%. These results suggest that although peripheral and central fatigue both influenced the fatigue-induced decrease in maximal muscle strength regardless of sex, the sex difference in KE fatigability is explained by that in peripheral fatigue, particularly the degree of peripheral VL fatigue.

## Introduction

An exercise-induced reduction in muscle force during maximal voluntary contraction (MVC) is defined as neuromuscular fatigue^1^. It can be quantified as a decline in maximal strength or the time to task failure of a sustained submaximal contraction. Although there is an exception^2^, women are considered to be less fatigable than men when performing specific similar-intensity isometric-fatiguing tasks^3^. Muscle fatigue depends on many peripheral and central factors^4^. Peripheral fatigue is shown as a decrease in force-generating capacity induced by processes occurring at or distal to the neuromuscular junction^5^. On the other hand, central fatigue is shown as a loss in neural drive or motor commands to the muscle, resulting in a reduction in force production or tension development^6^. Therefore, factors for the sex difference in muscle fatigability have been investigated from the viewpoint of peripheral and/or central fatigue^2,7–12^; however, it is unclear which of these factors more strongly influence the sex difference in muscle fatigability. Since human joint movement is produced by a number of muscles acting as synergists, a lack of detailed investigations on the degree of peripheral fatigue in each synergist is considered to be a reason for this ambiguity.

When muscles fail to completely relax during fatigue, resting tension develops^13,14^. Based on the theoretical model of muscle stiffness developed by Dresner *et al*.^15^, the greater resting tension results in the greater muscle stiffness. Therefore, the assumption that muscle fatigue induces an increase in resting muscle stiffness is reasonable. The resting muscle shear modulus is evaluated using shear wave ultrasound elastography as a muscle stiffness index^16,17^ and has been used to evaluate the degree of peripheral fatigue in each synergist before and after a fatiguing task^18^. Hence, as a previous study indicated^18^, the combination of evaluating muscle stiffness and traditional methods, such as surface electromyography (EMG) and evoked torque, may be helpful for more accurately investigating the difference in muscle fatigability between sexes than ever before.

The knee extensor muscles (KE) play an important role during locomotion such as walking and running. In this muscle group, the sex difference in fatigability was noted during low-level (20%-25% of maximal strength) sustained contractions^19–21^. A previous review^3^ has described that understanding the difference in fatigability between sexes is essential to know the limits of performance by KE in men and women and to establish optimal strategies for the training and rehabilitation of KE, relying on fatigability to provide adequate neuromuscular overload and ultimately neuromuscular adaptation and increased strength or endurance. If we can clarify the factors affecting the sex difference in fatigability during low-level (20%–25% of maximal strength) sustained contractions, the obtained findings are expected to be useful for proposing endurance training suitable for men and women. In the present study, we examined the shear moduli of KE before and after sustained low-level contractions using shear wave ultrasound elastography with other variables obtained by traditional approaches, such as surface EMG and evoked torque. As described above, a detailed investigation of the degree of peripheral fatigue in each synergist using shear wave ultrasound elastography may be useful for identifying which of the peripheral and central factors more strongly influence the sex difference in muscle fatigability. Therefore, the purpose of the present study was to investigate whether the difference in fatigue-induced increases in the shear modulus of one or more of the muscles of KE between the sexes may account for the sex difference in muscle fatigability.

## Results

### Variables before and after the fatiguing task

**Figure 1:**
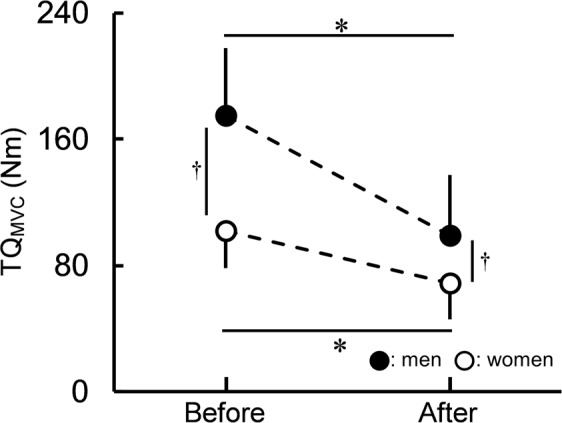
Peak torque (TQ_MVC_) for men and women before and after the fatiguing task. The time × sex interaction was significant. *Indicates a significant difference before and after the fatiguing task for each sex. ^†^Indicates a significant sex difference each before and after the fatiguing task. Data are presented as means ± standard deviation.

There was a significant time × sex interaction for peak torque during MVC (TQ_MVC_) (*P* < 0.001, partial η^2^ [η_p_^2^] = 0.464) (Fig. 1). Before (*P* < 0.001) and after (*P* = 0.004) the fatiguing task, TQ_MVC_ was significantly higher for men than for women (Fig. 1). TQ_MVC_ before the fatiguing task was significantly higher than TQ_MVC_ after the fatiguing task for men (*P* < 0.001) and women (*P* < 0.001) (Fig. 1). The percentage change in TQ_MVC_ showed a significantly greater decreased ratio of TQ_MVC_ induced by the fatiguing task for men (−44.5 ± 15.0%) than for women (−32.4 ± 16.8%) (*P* = 0.021, *r* = 0.359).

**Figure 2:**
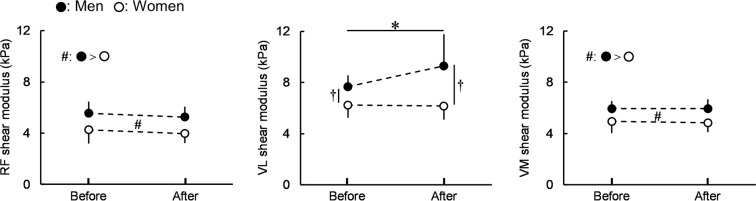
Shear moduli of the rectus femoris and lateral and medial vastus muscles (RF, VL, and VM) for men and women before and after the fatiguing task. Regarding RF and VM, there was no significant time × sex interaction. ^♯^Indicates the significant main effect of sex. Regarding VL, the time × sex interaction was significant. *Indicates a significant difference before and after the fatiguing task for men. ^†^Indicates a significant sex difference each before and after the fatiguing task. Data are presented as means ± standard deviation.

Figure 2 shows the shear modulus of each muscle. A significant time × sex interaction was found for the shear modulus of the lateral vastus muscle (VL) (*P* = 0.006, η_p_^2^ = 0.177). The fatiguing task-induced increase in the VL shear modulus was significant in men (*P* < 0.001), but not in women (*P* = 0.842). A significantly higher VL shear modulus for men than for women was observed before (*P* < 0.001) and after (*P* < 0.001) the fatiguing task. There was no significant time × sex interaction for the shear moduli of the rectus femoris muscle (RF) (*P* = 0.995, η_p_^2^ < 0.001) and medial vastus muscle (VM) (*P* = 0.581, η_p_^2^ = 0.008). The main effect of sex was significant (men > women) (RF: *P* < 0.001, η_p_^2^ = 0.423; VM: *P* < 0.001, η_p_^2^ = 0.416) without a significant main effect of time (RF: *P* = 0.051, η_p_^2^ = 0.094; VM: *P* = 0.747, η_p_^2^ = 0.003). Regarding percentage changes in the shear modulus of each muscle (RF: −3.4 ± 19.6% [men], −4.6 ± 18.6% [women]; VL: 21.7 ± 32.4% [men], 0.4 ± 20.4% [women]; VM: 1.6 ± 16.5% [men], −0.8 ± 14.2% [women]), there was a significant muscle × sex interaction (*P* = 0.024, η_p_^2^ = 0.092) and the percentage change in VL only was significantly higher for men than for women (*P* = 0.014). The percentage change in the VL shear modulus was significantly higher than those in the other muscle shear moduli for men (RF vs VL: *P* < 0.001; RF vs VM: *P* = 1.000; VL vs VM: *P* = 0.020), whereas no significant difference was observed in percentage changes in the shear moduli of muscles for women (RF vs VL: *P* = 0.922; RF vs VM: *P* = 1.000; VL vs VM: *P* = 1.000).

**Figure 3:**
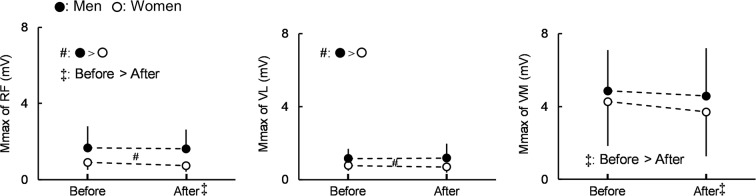
Peak-to-peak compound muscle action potential amplitude (Mmax) of the rectus femoris and lateral and medial vastus muscles (RF, VL, and VM) for men and women before and after the fatiguing task. There was no significant time × sex interaction for each muscle. ^♯^Indicates the significant main effect of sex. ^‡^Indicates the significant main effect of time. Data are presented as means ± standard deviation.

Regarding peak-to-peak compound muscle action potential amplitude (Mmax) (Fig. 3), there was no significant time × sex interaction in any muscle (RF: *P* = 0.265, η_p_^2^ = 0.032; VL: *P* = 0.159, η_p_^2^ = 0.050; VM: *P* = 0.394, η_p_^2^ = 0.019). The main effects of time (before > after, *P* = 0.027, η_p_^2^ = 0.120) and sex (men > women, *P* = 0.001, η_p_^2^ = 0.239) were both significant in the Mmax of RF. The significant main effect of sex (men > women, *P* = 0.006, η_p_^2^ = 0.178) was found in VL, while the main effect of time was significant (before > after, *P* = 0.018, η_p_^2^ = 0.135) in VM. A significant muscle × sex interaction (*P* = 0.679, η_p_^2^ = 0.010) and the main effects of muscle (*P* = 0.683, η_p_^2^ = 0.010) and sex (*P* = 0.221, η_p_^2^ = 0.038) were not found for percentage changes in the Mmax of the muscles (RF: −2.6 ± 19.5% [men], −11.4 ± 36.3% [women]; VL: 1.7 ± 34.2% [men], −10.6 ± 27.9% [women]; VM: −8.9 ± 29.9% [men], −10.7 ± 26.4% [women]).

**Figure 4:**
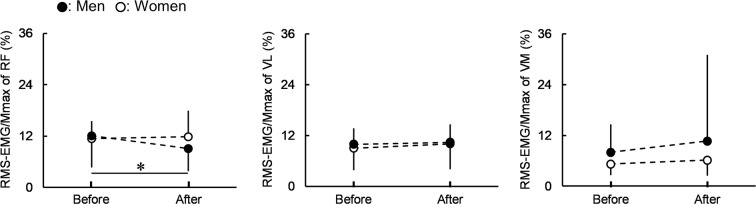
Root mean square values of surface electromyography signals normalized by peak-to-peak compound muscle action potential amplitudes (RMS-EMG/Mmax) of the rectus femoris and lateral and medial vastus muscles (RF, VL, and VM) for men and women before and after the fatiguing task. Regarding RF, the time × sex interaction was significant. *Indicates a significant difference before and after the fatiguing task for men. Regarding VL and VM, there were no significant main effects of time or sex without a significant time × sex interaction. Data are presented as means ± standard deviation.

Regarding root mean square value of electromyography signal (RMS-EMG) normalized by Mmax (RMS-EMG/Mmax) (Fig. 4), a significant time × sex interaction was only found in RF (*P* = 0.024, η_p_^2^ = 0.124). The RMS-EMG/Mmax of RF was significantly higher before than after the fatiguing task for men (*P* = 0.010), while the corresponding difference was not significant in women (*P* = 0.638). No significant sex difference was observed in the RMS-EMG/Mmax of RF regardless of before (*P* = 0.729) and after (*P* = 0.131) the fatiguing task. Regarding the RMS-EMG/Mmax of VL and VM, the main effects of time (VL: *P* = 0.262, η_p_^2^ = 0.032; VM: *P* = 0.281, η_p_^2^ = 0.030) and sex (VL: *P* = 0.666, η_p_^2^ = 0.005; VM: *P* = 0.204, η_p_^2^ = 0.041) were not significant. There was neither a significant muscle × sex interaction (*P* = 0.663, η_p_^2^ = 0.007) nor main effects of muscle (*P* = 0.083, η_p_^2^ = 0.070) and sex (*P* = 0.185, η_p_^2^ = 0.045) for percentage changes in the RMS-EMG/Mmax of muscles (RF: −21.1 ± 26.0% [men], 6.7 ± 41.5% [women]; VL: 8.3 ± 34.3% [men], 17.4 ± 47.2% [women]; VM: 12.5 ± 66.3% [men], 37.6 ± 129.9% [women]). Regarding the RMS-EMG of the biceps femoris muscle (BF) during knee extension normalized by that during knee flexion (nRMS-EMG) (before: 23.3 ± 21.3% [men], 27.1 ± 12.5% [women]; after: 20.7 ± 22.6% [men], 25.9 ± 15.1% [women]), there was no significant time × sex interaction (*P* = 0.609, η_p_^2^ = 0.007) or the significant main effects of time (*P* = 0.157, η_p_^2^ = 0.051) and sex (*P* = 0.415, η_p_^2^ = 0.017).

**Figure 5:**
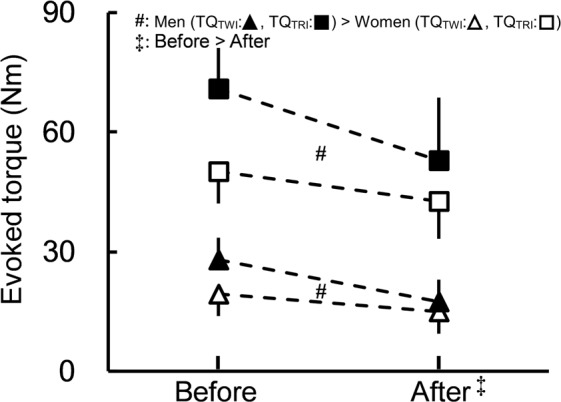
Evoked peak twitch and triplet torques (TQ_TWI_ and TQ_TRI_) for men and women before and after the fatiguing task. All of the first-order interactions (time × type, time × sex, type × sex) were significant without a significant second-order interaction (time × type × sex). In both types or both sexes, the evoked torques decreased significantly after the fatiguing task (‡). There were significant sex differences in the evoked torques (men > women: ♯) for both types or both times. Data are presented as means ± standard deviation.

Figure 5 shows evoked peak twitch and triplet torques (TQ_TWI_ and TQ_TRI_) before and after the fatiguing task. All of the first-order interactions (time × type [*P* < 0.001, η_p_^2^ = 0.297], time × sex [*P* = 0.001, η_p_^2^ = 0.234], type × sex [*P* < 0.001, η_p_^2^ = 0.351]) were significant without a significant second-order interaction (time × type × sex [*P* = 0.094, η_p_^2^ = 0.070]). In both types and both sexes, the evoked torques decreased significantly after the fatiguing task (all *P* < 0.001). There were significant sex differences in the evoked torques (men > women) for both types (all *P* < 0.001) and both times (before: *P* < 0.001; after: *P* = 0.022). The main effects of type (*P* < 0.001, η_p_^2^ = 0.441) and sex (*P* = 0.036, η_p_^2^ = 0.108) were both significant without a significant type × sex interaction (*P* = 0.255, η_p_^2^ = 0.033) for percentage changes in evoked torques (TQ_TWI_: −36.2 ± 23.7% [men], −22.1 ± 16.5% [women]; TQ_TRI_: −25.0 ± 20.9% [men], −14.8 ± 13.3% [women]), showing that the fatiguing task-induced decreased ratio of TQ_TWI_ was significantly greater than that of TQ_TRI_ and also that the corresponding decreased ratio was significantly greater for men than for women.

**Figure 6:**
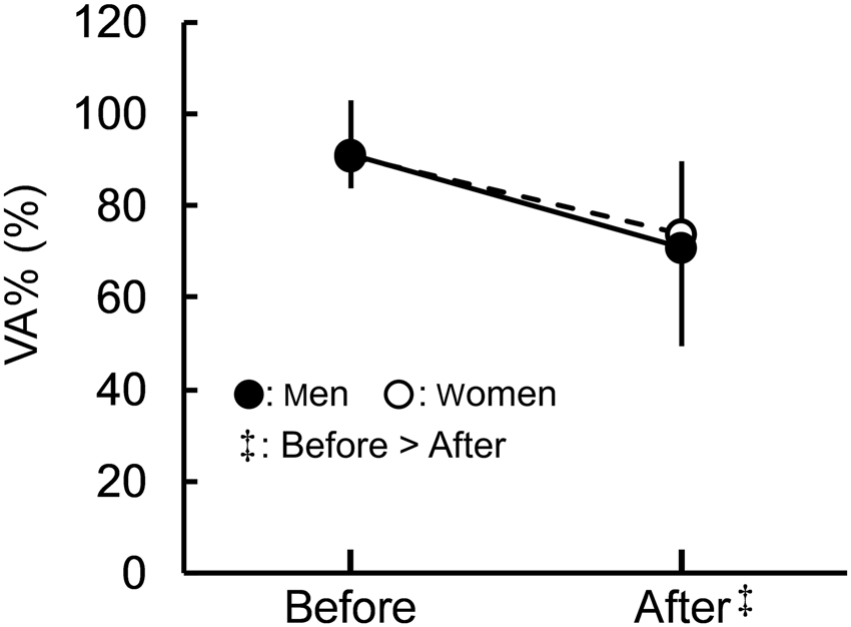
Voluntary activation (VA%) before and after the fatiguing task. A significant time × sex interaction was not found. ^‡^Indicates the significant main effect of time. Data are presented as means ± standard deviation.

Voluntary activation (VA%) before and after the fatiguing task are shown in Fig. 6. There was no significant time × sex interaction (*P* = 0.513, η_p_^2^ = 0.011) without the significant main effect of sex (*P* = 0.699, η_p_^2^ = 0.004), whereas the main effect of time was significant (before > after, *P* < 0.001, η_p_^2^ = 0.588). The percentage change in VA% was not significantly different between sexes (men: −22.6 ± 21.9%; women: −18.7 ± 13.9%) (*P* = 0.500, *r* = 0.109).

### Variables during the fatiguing task

The time to task failure was 393 ± 196 s for men and 481 ± 320 s for women. This sex difference was not significant (*P* = 0.313, *r* = 0.162).

Regarding normalized RMS-EMG during the fatiguing task (Table 1), only a first-order muscle × type interaction was significant with the significant main effect of sex (women > men). RMS-EMG during the former and latter halves of the fatiguing task normalized by RMS-EMG during maximal knee extension before the fatiguing task (%RMS-EMG_0–50_ and %RMS-EMG_50–100_) were significantly higher for VL than for RF and VM, and were significantly higher for RF than for VM. In each muscle, %RMS-EMG_0–50_ was significantly lower than %RMS-EMG_50–100_.

**Table 1:** Root mean square values of surface electromyography signals (RMS-EMG) of knee extensors during former and latter halves of the fatiguing task.

		%RMS-EMG_0–50_	%RMS-EMG_50–100_	*P* value	Effect size (Partial η^2^)
RF (%)	M	42.0 ± 16.3	51.5 ± 17.4	● interaction muscle × time × sex: 0.894 muscle × time: 0.008, muscle × sex: 0.121 time × sex: 0.803 ● main effect muscle: <0.001, time: <0.001, sex: 0.005 ● multiple comparison “muscle × time” RF vs VL: 0.006 (0–50), 0.001 (50–100) RF vs VM: 0.001 (0–50), 0.030 (50–100) VL vs VM: <0.001 (0–50), <0.001 (50–100) 0–50 vs 50–100: <0.001 (RF), <0.001 (VL), <0.001 (VM)	0.003 0.126, 0.053 0.002 0.402, 0.644, 0.181
	W	69.2 ± 43.7	77.1 ± 43.2		
VL (%)	M	56.5 ± 9.3	70.2 ± 14.9		
	W	79.7 ± 34.1	92.6 ± 35.9		
VM (%)	M	29.8 ± 10.7	44.0 ± 17.2		
	W	37.7 ± 15.2	52.0 ± 23.3		

Values are mean ± standard deviation.

%RMS-EMG_0–50_ and %RMS-EMG_50–100_, RMS-EMG during the former and latter halves of the fatiguing task normalized by RMS-EMG during maximal knee extension before the fatiguing task; RF, the rectus femoris muscle; VL, the lateral vastus muscle; VM, the medial vastus muscle; M, men; W, women.

The mean values of the joint torque level during the fatiguing task were 19.1 ± 0.5% (min: 18.6%, max: 20.3%) for men and 19.3 ± 0.5% (min: 18.3%, max: 20.4%) for women. The standard deviation (SD) of the joint torque level during the fatiguing task were 1.9 ± 0.4% (min: 1.3%, max: 2.5%) for men and 2.2 ± 0.7% (min: 1.2%, max: 4.4%) for women. The difference in the joint torque level during the fatiguing task between sexes was not significant (*P* = 0.246, *r* = 0.186). There was no correlation between the joint torque level during the fatiguing task and the percentage change in TQ_MVC_ in both sexes combined (*r* = 0.194, *P* = 0.224).

## Discussion

In the present study, a fatigue-induced decrease in TQ_MVC_ was found in men and women (Fig. 1), and the degree of the decrease in TQ_MVC_ induced by the fatiguing task was significantly greater for men than for women; however, there was no significant sex difference in the time to failure of the fatiguing task. That is, men were more fatigable than women when sustaining isometric knee extension at 20% of peak torque during MVC (20%MVC) until the endurance limit in the present study, which is consistent with previous findings^3^. TQ_MVC_ depends on peripheral and central factors^22^ and many variables related to peripheral or central factors changed significantly after the fatiguing task in the present study. Therefore, we would like to refer to factors for the sex difference in muscle fatigability based on the present results in as much detail as possible.

Unlike traditional approaches, the present study attempted to evaluate the degree of peripheral fatigue of each synergist using resting muscle shear moduli, as recently proposed^18^. As a result, the shear modulus only significantly increased for VL in men, not women after the fatiguing task (Fig. 2), and its percentage change from before to after the fatiguing task was significantly higher for men than for women. In addition, the percentage change in the VL shear modulus was significantly higher than those in the other muscle shear moduli for men; however, a corresponding difference was not found in women. These results suggest the more prominent degree of peripheral fatigue of VL for men after the fatiguing task, and may be explained by muscle activities during the fatiguing task or a difference in the muscle fiber composition of VL between the sexes. %RMS-EMG_0–50_ and %RMS-EMG_50–100_ were significantly higher for VL than for RF and VM (Table 1), which is generally consistent with previous findings^19^. Considering the present results and previous findings showing that VL is the largest muscle among the quadriceps femoris in both sexes^23^, it is reasonable that VL more strongly contributed to sustaining 20%MVC during the fatiguing task than the other muscles. In addition, the proportion of type II fibers in VL has been reported to be higher in men than in women^24^, and, thus, VL is considered to be more susceptible to fatigue in men than in women when sustaining isometric knee extension at 20%MVC although the present study did not investigate the fiber type composition of each KE for the participants directly. This may have resulted in the greater fatigue-induced increase observed in the VL shear modulus and higher VL activity (%RMS-EMG_0–50_ and %RMS-EMG_50–100_) during the fatiguing task, as shown in Table 1, for men than for women.

Regarding variables obtained by traditional approaches (i.e., variables except for muscle shear moduli), although peripheral and central fatigue induced by the fatiguing task were both observed, fatigue-induced changes in peripheral factors rather than central factors appeared to differ between the sexes in the present study. Evoked torques (TQ_TWI_ and TQ_TRI_), which are peripheral factors^25,26^, significantly decreased after the fatiguing task (Fig. 5). This result is consistent with the findings of many studies cited in a previous review^27^. On the other hand, regarding the present result showing that the decreased ratios of evoked torques were significantly higher for men than for women, previous findings are controversial. Some studies^9,10,12^ indicated no significant sex difference, whereas others^8,11^ found a greater relative reduction for men than for women. Differences exist in the exercise intensity (20%MVC, 80%MVC or MVC) or muscles examined (KE or the elbow flexors) between the present and previous studies. A previous study^2^ reported fatiguing task dependence on the mechanisms responsible for the sex difference in muscle fatigability. Unfortunately, among the above studies relevant to the sex difference in the fatigue-induced decreased ratio in evoked torques, none employed sustained isometric knee extension at 20%MVC. Therefore, it is difficult to simply compare the present results and previous findings of the corresponding sex differences and conclude this participant.

Regarding Mmax also reflecting peripheral fatigue^25,28,29^, the decrease induced by the fatiguing task was observed in RF and VM regardless of sex, but not in VL (Fig. 3). In accordance with a previous review^27^, whether Mmax decreases after the fatiguing task depended on the contraction duration. When this was shorter than 300 s, many studies showed no reduction in Mmax, whereas the twitch peak force and/or MVC force were reduced. In contrast, a decrease in Mmax and a concomitant reduction in twitch peak force and/or MVC force were noted when the contraction duration was longer than 600 s. The time to fatiguing task failure in the present study was generally between 300 and 600 s (men: 393 ± 196 s, women: 481 ± 320 s). Therefore, whether the fatiguing task-induced decrease in Mmax was observed appeared to vary with the muscles in the present study. A reduction in evoked torque/force after exercise is typically interpreted differently depending on the changes in Mmax^27^. When a decrease is noted in evoked torque/force with a similar reduction in Mmax after the fatiguing task, these changes are generally attributed to impaired neuromuscular propagation^30^. In contrast, a significant decrease in evoked torque/force with no/a minor change in Mmax may be affected by processes downstream of neuromuscular propagation, i.e., impaired Ca^2+^ handling and/or at the cross bridge level^31^. Based on these findings, the present results indicate a difference in sites in the periphery that are changed by fatigue between the muscles. However, no significant differences were observed in the percentage change in Mmax between the muscles or sexes; therefore, difficulties were associated with clearly distinguishing the aforementioned sites in the periphery between the muscles in the present study. In any case, the result of no sex difference in the percentage change in Mmax of each muscle suggests that the sex difference in fatigue-induced deceases in evoked torques cannot be explained by the results of Mmax.

Regarding RMS-EMG/Mmax^31,32^ and VA%^24^, which are indices for central fatigue induced by the fatiguing task, only the RMS-EMG/Mmax of RF for men was significantly increased (Fig. 4) and VA% for men and women was significantly reduced after the fatiguing task (Fig. 6). Thus, among the central factors, only the fatigue-induced change in the RMS-EMG/Mmax of RF showed a different trend between the sexes. However, no significant differences were noted in percentage changes in RMS-EMG/Mmax between the muscles and sexes, suggesting a smaller effect of the fatigue-induced change in the RMS-EMG/Mmax of RF on the sex difference in KE fatigability than some of the peripheral factors showing a sex difference in the degree of the change before and after the fatiguing task. Regarding VA%, many previous studies^8,9,12^ reported no sex difference in the degree of the fatigue-induced decrease in VA%, whereas one study^10^ demonstrated a significantly greater corresponding decline in men than in women. Consequently, the present results of no significant difference in the percentage change in VA% are consistent with previous findings.

Taken together, similar to the sex difference in muscle fatigability shown in the results obtained for TQ_MVC_, sex differences were observed in the degree of fatigue-induced changes in some of the peripheral factors (i.e., TQ_TWI_, TQ_TRI_, and the VL shear modulus). Based on traditional approaches, we suggest that stronger peripheral fatigue results in greater fatigability in men than in women. According to the results of muscle shear moduli in addition to traditional perspectives, the sex difference observed in the degree of peripheral fatigue of VL may have affected that in muscle fatigability in the present study.

In the present study, 20%MVC was used as the intensity of the fatiguing task, and the sex difference in muscle fatigability of KE was due mainly to the sex difference in the degree of peripheral fatigue of VL. Therefore, the findings of the present study can be applied to establish optimal training methods for improvement of KE fatigue resistance. For example, in the case of low-intensity endurance training for the purpose of improving KE fatigue resistance, the shorter training time seems to be required for young men than for young women to carry out the training efficiently and safely while reducing the fatigue of KE, especially VL.

The present study had some limitations. Firstly, changes in muscle blood volumes were not considered in the present study. Given that the shear moduli of RF and VM were not significantly increased after the fatiguing task (Fig. 2), the fatigue-induced changes in muscle blood volumes may affect the current results of muscle shear moduli somewhat. On the other hand, only the VL shear modulus for men increased after the fatiguing task (Fig. 2), with the higher percentage change in the VL shear modulus for men than for women. It is unlikely that the shear modulus increases dominantly for one sex, and the sex difference is attributed to a particular muscle among the synergists after the fatiguing task due to a change in the muscle blood volume. In other words, it is reasonable to assume that there was a negligible effect of a fatigue-induced change in the muscle blood volume on the interpretation of the present results for muscle shear moduli. Secondly, the distance between the innervation zone and surface EMG electrodes might have influenced the results of the present study slightly. We could not determine the corresponding effect in the current study; however, the increase of exercise intensity was accompanied by those of RMS values for each muscle and exercise regimen. Therefore, the aforementioned effect is considered to be small. Thirdly, the effect of individual differences in motivation was not considered in the current study. As shown in Fig. 6, both men and women had similar VA% before and after the fatiguing task. Based on these results, it is reasonable to assume that this effect on the interpretation of the current study was small. Fourthly, all measurements were performed with the participants’ right legs regardless of their leg dominance. In accordance with a previous study^33^, both dominant and non-dominant legs had similar endurance times during sustained isometric knee extension at 20%MVC although with a slight difference (approximately 5%) in MVC torque between legs. Moreover, in the current study, the participants completed a familiarization session to perform MVC and sustained 20%MVC of isometric knee extension in the first visit. Taken together, the fact that all participants performed contractions using the right leg is unlikely to have had a significant effect on the interpretation of the current results. Lastly, only the fatiguing task was used in the present study. As described above, the mechanisms responsible for the difference in muscle fatigability between sexes have been suggested to be task dependent^2^. Hence, it is unclear whether the present results may be generalized to other intensities or contraction types for KE. Given our previous findings^18^ and the current results, the use of the muscle shear modulus is expected to be useful for deepening our understanding of the mechanisms of muscle fatigue and its sex difference under other conditions. Further studies are needed to examine the aforementioned question using the muscle shear modulus assessed using ultrasound shear wave elastography.

In summary, the present study examined the shear moduli of KE before and after sustained low-level (20%MVC) knee extension until the endurance limit using shear wave ultrasound elastography with other factors obtained by traditional approaches (i.e., surface EMG and evoked torque). The decrease in maximal muscle strength induced by the fatiguing task was more prominent in men than women. Of the KE shear moduli, only the VL shear modulus for men increased after the fatiguing task, and a difference in the percentage change from before to after the fatiguing task between sexes was noted in the VL shear modulus. In addition, evoked torques (TQ_TWI_ and TQ_TRI_) also decreased after the fatiguing task with decreased ratios being greater for men than for women. On the other hand, a fatigue-induced decrease in VA% was noted in men and women, but the decreased ratio of VA% was not significantly different between the sexes. These results suggest that although peripheral and central fatigue both influenced fatigue-induced decreases in maximal muscle strength regardless of sex, the difference in the fatigue-induced decline in maximal muscle strength between the sexes was due mainly to the sex difference in the degree of peripheral fatigue of VL.

## Methods

### Participants

After providing written informed consent, 41 healthy young men (n = 18; age: 22 ± 1 yr, height: 172.2 ± 5.2 cm, body mass: 63.5 ± 8.2 kg; mean ± SD) and women (n = 23; age: 21 ± 1 yr, height: 159.0 ± 5.0 cm, body mass: 50.9 ± 5.6 kg; mean ± SD) voluntarily participated in this study. They were free of cardiovascular diseases, sedentary, and did not perform any regular exercise. All measurements were performed with the participants’ right legs. This study was approved by the Ethics Committee of the Shibaura Institute of Technology and conducted according to the Declaration of Helsinki.

### Experimental procedures

Participants visited our laboratory twice. In the first visit, participants completed a familiarization session to perform maximal voluntary contractions (MVC) and low-level sustained contractions of isometric knee extension. They sat on the seat of a dynamometer (CON-TREX MJ, Physiomed, Germany) with the hip at 80° and the knee at 90° of flexion (anatomical position = 0°). The participant’s pelvis, torso, and ankle were secured on the reclining seat and dynamometer with non-elastic straps and/or a seat belt. Care was taken to adjust the centers of rotation of the knee joint and dynamometer. After conducting several submaximal knee extension contractions as a warm-up, participants performed MVC for 3 s two times with a 1-min interval. If the difference in peak torque between the two contractions was more than 10%, the peak torque was measured one more time. The highest value of two or three peak torque measurements was used to calculate 20%MVC. Participants then sustained an isometric contraction at 20%MVC for approximately 20 s two or three times with a 1-min interval. Torque signals were recorded at a sampling frequency of 2000 Hz and stored in a personal computer using LabChart software (v8.1.5, ADInstruments, Australia) after the A/D conversion (PowerLab16/35, ADInstruments, Australia). During the sustained 20%MVC, torque data were displayed as waveforms on the monitor of a personal computer using LabChart software in real time. The monitor was placed in front of the participants to provide visual feedback of torque level to participants in real time. The horizontal target line (20%MVC) was displayed on the monitor using the software, and participants attempted to match their torque level and target line.

The actual measurement was conducted in the second visit. Participants sustained isometric knee extension at 20%MVC until the endurance limit as the fatiguing task. Before and after this task, the shear moduli, surface EMG signals, and peak-to-peak compound muscle action potential amplitude (Mmax) of RF, VL, and VM as well as evoked and voluntary peak torques, including VA% of KE, were assessed.

Participants stood with their arms relaxed at their sides and the right thigh leg length from the greater trochanter to the popliteal crease was measured to the nearest 0.5 cm with a steel tape. Participants then sat on the seat of the dynamometer (CON-TREX MJ, Physiomed, Germany) as described previously, and the measurement sites of EMG signals and the shear wave propagation speeds used to calculate muscle shear moduli were identified. The measurement sites of the shear wave propagation speeds within RF, VL, and VM were set at the distal 70%, 50%, and 30% of the thigh length, respectively. Regarding mediolateral directions, both ends of each muscle were confirmed using a B-mode ultrasound apparatus (ACUSON S2000, Siemens Medical Solutions, USA) with a 45-mm electronic linear array probe (9L4 Transducer, 4–9 MHz, Siemens Medical Solutions, USA) and the midpoints of each muscle were identified as the measurement sites. The electronic linear array probe (9L4 Transducer) was longitudinally placed and its direction was basically adjusted to match the orientation of the muscle fascicle at each measurement site. The positions at which the electronic linear array probe was placed were marked with a pen. The measurement sites of the EMG signals of RF, VL, VM, and BF were set at the distal 50%, 70%, 20% and 50% of the thigh length in the proximodistal direction, respectively, and at the midpoints of both ends of each muscle in the mediolateral direction confirmed using ultrasonography. Before marking the positions of each pre-amplified bipolar surface electrode (1 × 10 mm, inter-electrode distance of 10 mm; DE-2.1, DELSYS, USA) with 20–450 Hz band-pass filter (The Bagnoli 8 EMG System, DELSYS, USA), the orientation of the muscle fascicle at each measurement site was confirmed using ultrasonography to match the electrode direction. The reference electrode was planned to be placed over the left medial malleolus. The electrodes were placed at each site after skin shaving, rubbing with sandpaper, and cleaning with alcohol.

**Figure 7:**
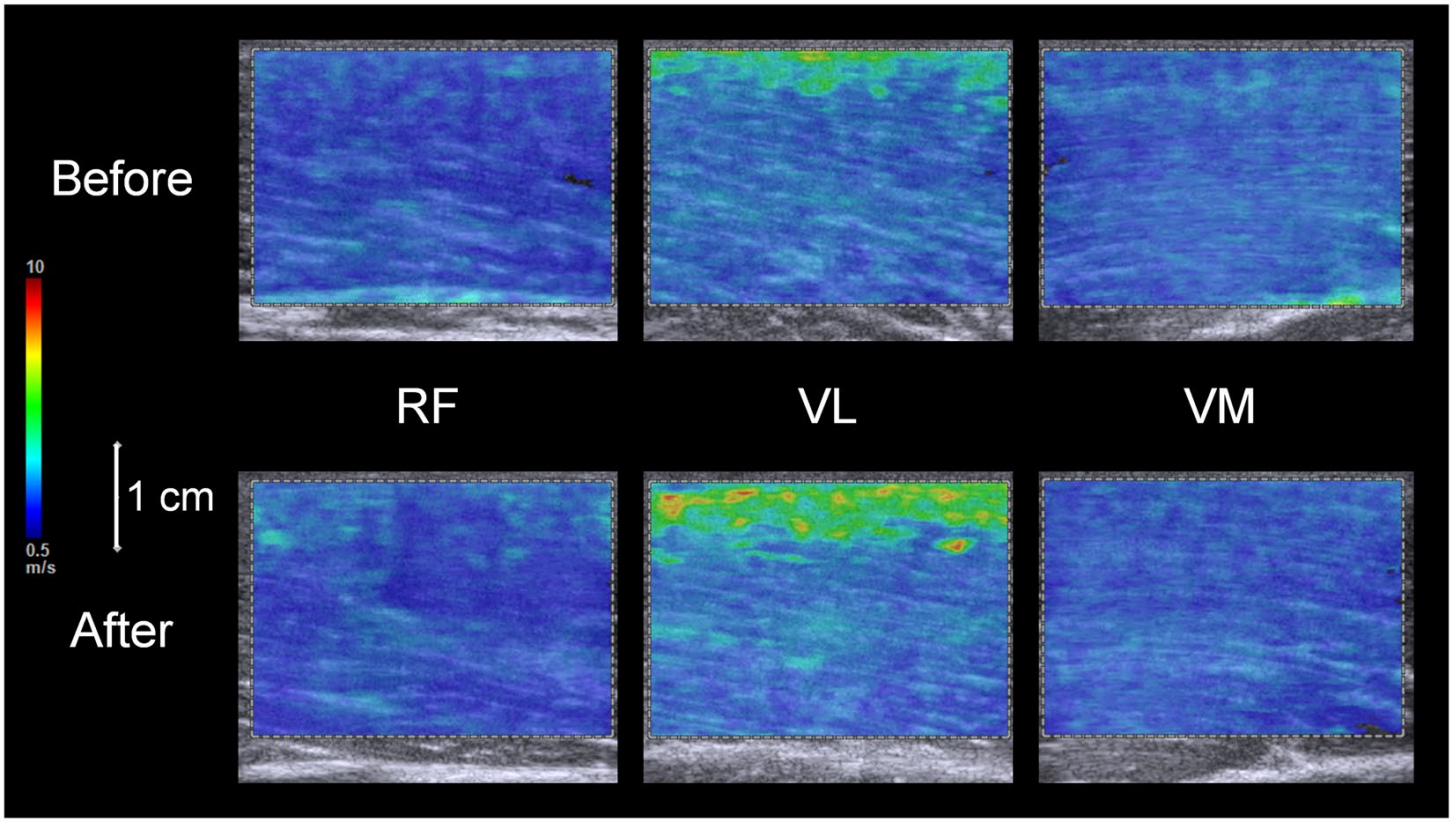
Typical elastography images of the rectus femoris and lateral and medial vastus muscles (RF, VL, and VM) before (upper) and after (lower) the fatiguing task. Shear wave ultrasound elastography generated color-coded images with a scale from blue (soft) to red (hard) depending on the magnitude of the shear wave propagation speed.

The participant’s torso, pelvis, and ankle were then secured on the dynamometer and reclining seat with a seat belt and non-elastic straps, and shear wave propagation speeds within the resting RF, VL, and VM before the fatiguing task were measured three times in a random order (Fig. 7). The electronic linear array probe was prepared with water-soluble transmission gel and was placed at the measurement site without depression of the tissues. An elastographic image with a color map of shear wave propagation speed (color-coded area [depth × width]: 2.5 × 3.5 cm) was acquired once. This image was examined to see whether the shear wave was sufficient in quality to produce accurate measurements of shear wave propagation speeds within a color map using a function of the ultrasound apparatus; Green and orange pixels indicate high and low quality, respectively. We repeated the measurements before three elastography images with sufficient quality were considered to be obtained.

Muscle shear propagation speed measurements were followed by assessments of evoked and voluntary peak torques and Mmax. In order to percutaneously stimulate the femoral nerve, a cathode (2 × 2 cm) was set in the femoral triangle and anode (4 × 5 cm) was placed midway between the superior aspect of the greater trochanter and inferior border of the iliac crest^34^, and a constant-current variable voltage stimulator (DS7A, Digitimer Ltd., UK) and a controller (SEN-3401, Nihon Kohden, Japan) were used. Stimulus intensity was gradually increased until a plateau in twitch torque was reached, and supramaximal stimulus intensity was set at 1.2 times the above stimulus intensity for experimental measurements to ensure maximal muscle activation^35,36^. Each of resting evoked twitch and triplet responses was obtained twice every 10 s. After performing several submaximal knee extension contractions as a warm-up, the MVC peak torque was measured two times with a 1-min interval. This measurement was performed one more time when the difference between the first two values of peak torque was >10% of the higher one. The highest value of two or three measurements was adopted as TQ_MVC_ to calculate the target level during the fatiguing task (i.e., 20%MVC). In addition, the peak torque during knee flexor MVC was also measured two or three times with a 1 min interval. Similar to torque signals, EMG signals were recorded at a sampling rate of 2000 Hz and were stored in a personal computer using LabChart software (v8.1.5, ADInstruments, Australia) after the A/D conversion (PowerLab16/35, ADInstruments, Australia). During TQ_MVC_ measurement, investigators provided strong verbal encouragement to the participants so that they performed knee extension with their maximum effort.

A practice session for sustaining 20%MVC was subsequently conducted. Participants were instructed to perform 6 isometric knee extensions at 20%MVC for approximately 10 s^37^ with a 1-min interval. As described in the explanation given on the first visit, visual feedback of torque data was provided in real time to participants, and they attempted to match the horizontal target line (20%MVC) and their torque level. After a 10-min rest period, participants performed isometric MVC of KE for 3 s four or five times. In the last two MVC performances, supramaximal triplet stimulations were interpolated at 100 Hz during and after MVC in order to assess VA%. Of the four or five peak torques, the highest value was confirmed to be similar to TQ_MVC_ before the practice session for each participant (1.8 ± 8.8%).

Participants took a 5-min rest and started the fatiguing task. As described in the previous paragraph, participants attempted to match their torque level and the horizontal target line (20%MVC) as long as possible. Task failure was defined as the instant when torque fell below another horizontal line showing 15%MVC, which was also displayed on the monitor using the software, for longer than 5 s. Although participants and investigators were not aware of the contraction time, investigators provided strong verbal encouragement to participants during the fatiguing task, especially while the torque level remained below 15%MVC for a few seconds. Task failure was judged by the investigator, and the participants stopped the contraction at this time point.

Immediately following the termination of the fatiguing task, one resting evoked twitch and triplet responses were obtained every 10 s. After a 10-s rest period, TQ_MVC_ was measured once. After 30 s of the TQ_MVC_ measurement, supramaximal triplet stimulations at 100 Hz were interpolated during and after MVC in order to evaluate VA%. Immediately after that, shear wave propagation speeds within each muscle were measured three times, in the same order of the measurement before the fatiguing task.

### Data analyses

Shear wave propagation speeds within RF, VL and VM were assessed using image analysis software (MSI Analyzer version 2.0Aql, Institute of Rehabilitation Science, Tokuyukai Medical Corporation, Japan). A quadrangular region of interest (ROI) was set on the muscle to be as large as possible within the color-coded area of the elastography image, and the mean value of the shear wave propagation speed within the ROI was automatically obtained at 0.01 m/s. Analyses of each image were conducted once. The mean values of the three measurements were used for further analyses. The coefficients of variance (CVs) and intraclass correlation coefficients (ICCs) type 1,3 for these measurements were 2.4 ± 2.0% and 0.974 (*P* < 0.001) for RF, 2.3 ± 2.0% and 0.980 (*P* < 0.001) for VL, and 1.6 ± 1.8% and 0.975 (*P* < 0.001) for VM. In addition, the day-to-day repeatability of the shear propagation speed within each muscle before the fatiguing task was evaluated in 9 young men. The CVs of the two measured values were 3.5 ± 3.2% for RF, 1.8 ± 2.3% for VL, and 2.4 ± 2.2% for VM.

The shear modulus of a muscle is calculated as the product of muscle density and shear wave propagation speed squared^18^. In the present study, the muscle density was assumed to be 1084 kg/m^3^, which was the mean of the two values (1112 and 1055 kg/m^3^) obtained from different methods in a cadaveric study^38^.

Of the two evoked peak twitch and triplet torques before the fatiguing task, the higher values were adopted as TQ_TWI_ and TQ_TRI_ before the fatiguing task, respectively. In addition to TQ_TWI_, the Mmax of each muscle was measured during the resting evoked twitch response. The two values of the Mmax of each muscle before the fatiguing task were averaged for further analyses.

The RMS-EMGs during MVC were evaluated over a 0.5-s period around the peak torque. Before the fatiguing task, these values in the selected task in which the highest value of the peak torque was observed were used. RMS-EMGs were normalized by Mmax (RMS-EMG/Mmax) in RF, VL, and VM both before and after the fatiguing task. The RMS-EMG of BF during knee extension was normalized by that during knee flexor MVC in the selected task in which the highest value of peak torque was observed before the fatiguing task (nRMS-EMG).

The calculation of RMS-EMGs of RF, VL, and VM during the fatiguing task was divided into two parts: during the former and latter halves of the sustained contraction. In each muscle, the values of RMS-EMGs during the former and latter halves of the sustained contraction were normalized using RMS-EMG in the selected MVC task before the fatiguing task (%RMS-EMG_0–50_ and %RMS-EMG_50–100_).

VA% was assessed using the following formula: VA% = (1 – [superimposed triplet torque/potentiated resting triplet torque]) × 100 (%). Before the fatiguing task, the two values of VA% were averaged for further analyses.

TQ_MVC_ depends on peripheral and central factors^22,26^. In the present study, fatiguing task-induced changes in the shear modulus^18^ and Mmax^25,28,29^ of each muscle, TQ_TWI_^25^, and TQ_TRI_^26^ were considered to reflect peripheral fatigue. On the other hand, central fatigue was evaluated using fatiguing task-induced decreases in RMS-EMG/Mmax^31^ and VA%^24^.

### Statistical analyses

A two-way analysis of variance (ANOVA) with one within-group factor (time [before and after the fatiguing task]) and one between-group factor (sex [men and women]) was used to evaluate changes induced by the fatiguing task in the shear modulus, Mmax and RMS-EMG/Mmax of each muscle, nRMS-EMG of BF, TQ_MVC_, and VA%. When a significant interaction was detected, additional ANOVA with the Bonferroni multiple-comparison test was performed. A three-way ANOVA with two within-group factors (time [before and after the fatiguing task], type [TQ_TWI_ and TQ_TRI_]) and one between-group factor (sex [men and women]) was used to evaluate fatiguing task-induced changes in evoked torques. When a significant first-order interaction was detected without a significant second-order interaction, the Bonferroni multiple-comparison test was performed as additional ANOVA.

Percentage changes in the shear moduli, Mmax and RMS-EMG/Mmax of each muscle, TQ_MVC_, TQ_TWI_, TQ_TRI_, and VA% from before to after the fatiguing task were also calculated. Differences in percentage changes in the shear moduli and RMS-EMG/Mmax among the muscles were examined using a two-way ANOVA with one within-group factor (muscle [RF, VL and VM]) and one between-group factor (sex [men and women]) followed by the Bonferroni multiple-comparison test. Similarly, percentage changes in the evoked torques were also evaluated using a two-way ANOVA with one within-group factor (type [TQ_TWI_ and TQ_TRI_]) and one between-group factor (sex [men and women]) followed by the Bonferroni multiple-comparison test. Sex differences in percentage changes in TQ_MVC_ and VA% were examined using an unpaired *t*-test.

Regarding normalized RMS-EMG during the fatiguing task, a three-way ANOVA with two within-group factors (muscle [RF, VL and VM] and type [%RMS-EMG_0–50_ and %RMS-EMG_50–100_]) and one between-group factor (sex [men and women]) was used. When a significant first-order interaction was detected without a significant second-order interaction, additional ANOVA with the Bonferroni multiple-comparison test was conducted. The sex difference in the time to task failure was examined using an unpaired *t*-test.

In order to test the effects of individual variability in joint torque levels during the fatiguing task on fatigue-induced decreases in joint torque, joint torque data during the fatiguing task were initially normalized by TQ_MVC_ as the joint torque level during the fatiguing task. The sex difference in the joint torque level during the fatiguing task was then examined using an unpaired *t*-test. Furthermore, Pearson’s product-moment correlation coefficient was calculated between the joint torque level and percentage change in TQ_MVC_ from before to after the fatiguing task in both sexes combined.

Data are presented as means ± SDs. Statistical significance was set to *P* < 0.05. When the results of the unpaired *t*-test or those of the main effect and interaction of the two-way ANOVA and three-way ANOVA are presented, *r* or η_p_^2^ are shown as indices of the effect size with the *P* value. Statistical analyses were performed using statistical analysis software (SPSS 25.0, IBM, USA).
